# Effect of Lignocaine and a Topical Vapocoolant Spray on Pain Response during Surgical Castration of Beef Calves

**DOI:** 10.3390/ani9040126

**Published:** 2019-03-28

**Authors:** Dominique Van der Saag, Sabrina Lomax, Peter Andrew Windsor, Evelyn Hall, Peter John White

**Affiliations:** 1 Sydney School of Veterinary Science, Faculty of Science, The University of Sydney, Sydney, NSW 2006, Australia; peter.windsor@sydney.edu.au (P.A.W.); evelyn.hall@sydney.edu.au (E.H.); p.white@sydney.edu.au (P.J.W.); 2 School of Life and Environmental Sciences, Faculty of Science, The University of Sydney, Sydney, NSW 2006, Australia; sabrina.lomax@sydney.edu.au

**Keywords:** behaviour, castration, cattle, lignocaine, pain, ocular temperature, stress, vapocoolant spray

## Abstract

**Simple Summary:**

Ensuring a good state of welfare in farm animals is important for ethical reasons and sustainability of livestock industries. Addressing pain in farm animals during and following surgical husbandry procedures is a significant component of animal welfare. Until recently, practical constraints associated with delivery of traditional forms of analgesia have prevented widespread uptake by producers, especially in large, extensively managed animal production systems. In recent years, novel anaesthetic and analgesic products have been developed to facilitate practical delivery of post-operative pain relief to livestock. However, the issue of intra-operative pain during husbandry procedures remains unaddressed. Therefore, this study investigated the efficacy of pre-operative injected lignocaine and peri-operative topical vapocoolant spray, administered as most practical for incorporation into routine procedures, for pain relief during castration of calves. Results from this study provide no evidence that lignocaine or vapocoolant spray reduced pain during castration of calves. Pulling of the spermatic cords appeared to be the most painful component of the castration procedure. This finding may assist in clarifying what methods for relieving procedural pain associated with castration of calves merit future research.

**Abstract:**

This study assessed the efficacy of pre-operative injected lignocaine and peri-operative topical vapocoolant spray, administered as most practical for incorporation into routine calf castration procedures. Calves were randomly allocated to: (1) sham castration (SHAM); (2) surgical castration (CAST); (3) surgical castration with lignocaine (LIG); and (4) surgical castration with vapocoolant spray (VAPO). Calf behavioural responses were scored at different stages of the sham castration or castration procedure. Maximum ocular temperatures were measured at three time-points relative to restraint and treatment. There were significant effects of treatment (*p* < 0.001) and stage of procedure (*p* < 0.001) on calf behavioural response. SHAM calves were more likely to display less severe responses compared to all other calves and LIG calves were more likely to display less severe responses compared to VAPO calves. Calves were more likely to display more severe responses to extrusion of the first spermatic cord compared to all other stages of castration, and to extrusion of the second spermatic cord compared to severing of the second spermatic cord. There was a significant effect of time (*p* < 0.001) on ocular temperature, with ocular temperature being greater following sham castration or castration. In this study, there was no evidence of pain reduction during castration of calves by either lignocaine or vapocoolant spray.

## 1. Introduction

Castration of beef cattle is performed to prevent unwanted breeding, improve meat quality and reduce aggressive behaviour. In Australia, the most common methods to castrate calves are surgical cutting or rubber ring application, both of which cause pain [1]. For large commercial beef operations in northern Australia, time associated with additional animal handling is a major constraint to the use of traditional forms of anaesthesia and analgesia [2]. In recent years, advancements to post-operative pain relief for calves undergoing castration have been demonstrated through the development of practically administered analgesic products, such as a topical anaesthetic, Tri-Solfen^®^ (Bayer Australia Ltd, Pymble, NSW, Australia) and a buccal non-steroidal anti-inflammatory drug, Ilium^®^ Buccalgesic OTM (Troy Laboratories Australia Pty Ltd, Glendenning, NSW, Australia). Such products can be quickly and easily administered by producers whilst calves are in a race, crush or cradle [3,4,5,6]. However, practical options to address intra-operative pain associated with surgical castration are lacking.

Local anaesthetics can be used to penetrate nerves and block impulse conduction by inhibiting voltage-gated sodium channels for a duration of 60 to 120 min [7]. Lignocaine is the local anaesthetic most commonly used in cattle [8], as it has a faster onset of action (5 to 10 min) than most other local anaesthetics [7]. Injecting lignocaine, either locally or as an epidural nerve block, is a commonly accepted method for providing pain relief during and in the acute period following castration of calves [8]. The double handling or longer calf restraint time required for administration of injected local anaesthetic can be viewed as impractical and detrimental to animal welfare [2] and is likely the main reason that this method of pain relief has not been widely adopted by producers.

Topical vapocoolant sprays have been used in recent years to provide local anaesthesia prior to injections and minor surgical procedures in humans [9]. They have been shown to decrease pain associated with initial intradermal anaesthetic injection [10], venipuncture [11], vaccinations [12], cosmetic botulinum injections [13] and intravenous cannulation [14]. Vapocoolant sprays have also been shown to reduce pain responses in horses during arthrocentesis [9] and in calves during ear tagging and ear notching [15]. Topical vapocoolants result in local anaesthesia by causing a drop in temperature due to rapid evaporation of volatile liquid from the skin surface [10]. This temporary epidermal hypothermia interrupts initiation and conduction of neural impulses in local sensory nerves, resulting in reduced pain sensation [14]. Vapocoolant spray could be a practical option for producers to provide pre-operative anaesthesia, as it is low cost, easily administered, immediately effective [16] and painless [14].

The aim of this study was to assess and compare the efficacy of pre-operative injected lignocaine and peri-operative topical vapocoolant spray, administered as most practical for incorporation on-farm, to provide local anaesthesia during surgical castration of calves. We hypothesised that lignocaine and vapocoolant spray would reduce pain-related responses of calves during surgical castration. The greater aim of this research is to identify a practical analgesic regime that addresses all stages of the pain response to castration in calves, based on findings from previous, current and future studies.

## 2. Materials and Methods

### 2.1. Animals

The experimental protocol was approved by the Animal Ethics Committee of the University of Sydney (approval number 5832). Forty unweaned Angus bull calves (2–4 months old), weighing 103.5 ± 30.2 kg, were randomly selected for the trial from a commercial herd on a University of Sydney farm in the Southern Tablelands of NSW, Australia. Surgical castration is undertaken as a routine farm management procedure at this property. Calves were routinely ear tagged and ear notched one week prior to the trial.

### 2.2. Experimental Design and Treatments

On the day of the trial, calves were separated from their mothers and held for 1 h in a yard adjacent to the cattle race. The calves were quietly moved through the race towards a calf cradle where they were weighed using cattle scales, W810 (Gallagher Group Ltd, Hamilton, Waikato, New Zealand) within the race and then restrained in right lateral recumbency in a calf cradle (Arrow Farmquip, Tamworth, NSW, Australia) for treatment and data collection.

The calves were randomly allocated to one of four treatments: (1) sham castration (SHAM, *n* = 10); (2) surgical castration (CAST, *n* = 10); (3) surgical castration following pre-operative injections of local anaesthetic lignocaine (Ilium Lignocaine 20^®^, Troy Laboratories, Glendenning, NSW, Australia) (LIG, *n* = 10); and (4) surgical castration following peri-operative applications of topical vapocoolant spray (Animal Ethics Pty Ltd, Yarra Glen, Vic, Australia) comprising a hydrocarbon propellant in an aerosol canister (VAPO, *n* = 10).

The sham castration treatment group was included to control for possible temporal effects of restraint and non-injurious physical manipulation of the scrotum and testicles. For SHAM calves, testes were gently physically manipulated for 30 s with no surgical intervention. Surgical castration was performed by transversely excising the distal third of the scrotum with a sharpened sterilised knife, followed by application of downward pressure to the scrotum above the testicle-enabled extrusion of each testis and spermatic cord from the tunica vaginalis. Following extrusion, each spermatic cord was severed with the knife using a scraping motion. The castration procedure took approximately 30 s. For LIG calves, 3 mL of lignocaine HCl (20 mg/mL) were injected into each side of the scrotum and a further 3 mL into each spermatic cord, using a 10 mL syringe and an 18 G needle, 5 min prior to castration. The relatively short duration between administration of lignocaine and castration (5 min) was purposely chosen with consideration of practical limitations associated with adding too much time onto the entire operation. In addition, animal welfare concerns associated with longer calf restraint times were considered. For VAPO calves, vapocoolant spray was applied to the distal scrotum immediately prior to excision and then to each exposed spermatic cord following extrusion, prior to severing. Vapocoolant spray was applied for 3 s from a distance of 10 cm to the scrotum and spermatic cords, as this application method has been shown to be effective for ear tagging and notching [15]. In addition, this application method is suitable for an animal production setting where a short spray duration (3 s) does not add substantial time to animal processing [15]. In the current study, application of vapocoolant spray added approximately 9 s onto the castration procedure.

### 2.3. Behavioural Scoring

A video camera mounted on a tripod was used to film the behavioural responses of castrated calves to the procedures. Ear tag numbers were visible in the videos, allowing for individual animal identification. Each video was later scored individually by two trained observers. There were two scores that differed between observers. These scores were reassessed following discussion, resulting in agreeance between both observers for all scores. Behavioural responses were scored on a numerical rating scale of 0 to 3, taken from previous studies on the behavioural response to ear tagging and ear notching [15] and castration [17,18,19,20,21] of calves and were as follows: 0 = no movement; 1 = mild movement (mild head and/or body movement, including ear and/or tail flick, wince or nasal flare); 2 = moderate movement (moderate head and/or body movement, including head shake, twisting, mild kicking and mild vocalisation); and 3 = severe movement (severe head and/or body movement including kicking, full head movement from cradle and severe escape response, bellowing). An individual score was assigned to each consecutive stage of the castration procedure: (1) excision of scrotum; (2) extrusion of right spermatic cord; (3) severing of right spermatic cord; (4) extrusion of left spermatic cord; and (5) severing of left spermatic cord. For SHAM calves, scores for each of these stages were assigned at 6-second intervals throughout the sham castration procedure. This resulted in a total of five scores for each calf.

### 2.4. Ocular Temperature

Infrared photographs of the left eye were captured from calves using a handheld infrared camera, FLIRE50 (FLIR Systems Australia Pty Ltd, Mulgrave, Vic, Australia), with a thermal range of −20 °C to 120 °C and a sensitivity of 0.045 °C. Infrared photographs were taken at two time-points; immediately following restraint in a calf cradle and immediately following castration. A cardboard frame, with 10 cm by 10 cm dimensions, was used to standardise the image area by holding it over the eye with the eye in the centre. The camera frame was then aligned with the cardboard frame for each photograph. This ensured the camera lens was at a consistent distance of 0.5 m from the eye. This distance, along with an emissivity value of 0.95 were entered into the infrared camera for calibration. Ambient temperature was monitored and entered into the infrared camera for calibration every 30 min during the data collection period. In addition, ambient temperature was recorded at the time each photograph was captured. Images were analysed for maximum temperature using thermal imaging program FLIR Tools Software (FLIR Systems Australia Pty Ltd, Mulgrave, Vic, Australia). This software allowed for analysis of a specific area using a geometric figure drawn on the photograph. A circle figure was drawn around the eye in each photograph and the maximum temperature within this area was calculated.

### 2.5. Statistical Analysis

#### 2.5.1. Behavioural Scoring

Behavioural score data were subjected to ordinal logistic regression (OLR) in ASReml 3.0 statistical software (VSN International, Hemel Hempstead, Hertfordshire, UK). The fixed effects considered for inclusion in the model were treatment (SHAM, CAST, LIG, VAPO), stage of procedure (1 to 5) and their interaction and body weight (variate). CalfID was included as a random effect in the model. Insignificant terms were dropped from the model using a backwards elimination approach until all terms in the final model were significant. *p* values ≤ 0.05 were considered statistically significant. Post-hoc pair-wise comparisons using least significant differences at a level of *p* ≤ 0.05 were conducted to analyse differences between groups. Data are presented as cumulative probabilities of calves displaying behavioural response scores of *Y* = 0, 1, 2 and 3. Individual odds ratios were generated for significant factors in R 3.4.3 statistical software (R Foundation for Statistical Computing, Vienna, Austria).

#### 2.5.2. Ocular Temperature

Maximum ocular temperature data was subjected to restricted maximum likelihood (REML) for repeated measures using the mixed models procedure of Genstat 17th Edition statistical software (VSN International Ltd, Hemel Hempstead, Hertfordshire, UK). The fixed effects considered for inclusion in the model were treatment (SHAM, CAST, LIG, VAPO), time-point (1, 2) and their interaction, body weight (variate) and ambient temperature (variate). CalfID was included as a random effect in the model. Insignificant terms were dropped from the model using a backwards elimination approach until all terms in the final model were significant. Significant variates were subjected to a Spearman’s rank correlation with maximum ocular temperature using the nonparametric correlations procedure of Genstat. *p* values ≤ 0.05 were considered statistically significant. Data are presented as predicted means (± standard error of the mean).

## 3. Results

### 3.1. Behavioural Scoring

**Figure 1:**
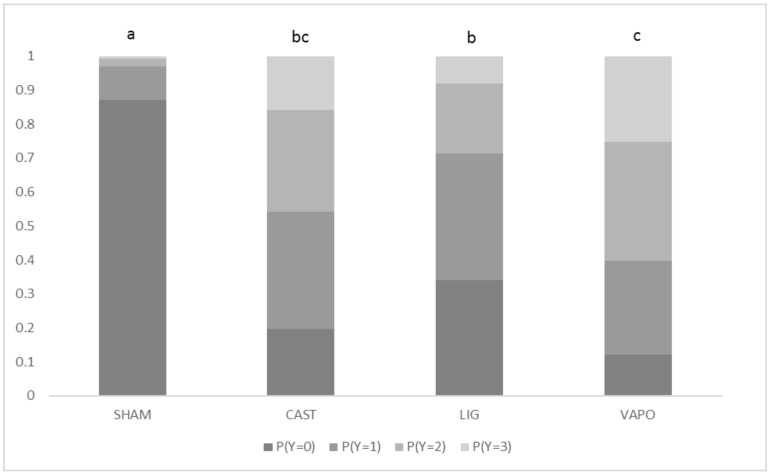
**Probability (P) of calves in each treatment group displaying behavioural response scores (Y; 0 = zero movement, 1 = mild movement, 2 = moderate movement, 3 = severe movement) to sham castration or castration.** Treatment: SHAM = sham castrated; CAST = castrated; LIG = castrated and treatment with local anaesthetic lignocaine; and VAPO = castrated and treatment with vapocoolant spray. ^a,b,c^ Treatments with different superscripts differ significantly at *p* ≤ 0.05. A significant effect was found (*p* < 0.001).

**Figure 2:**
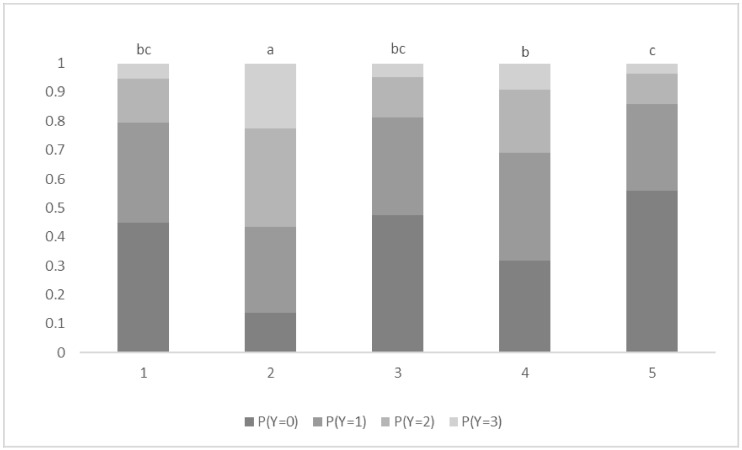
**Probability (P) of all calves displaying behavioural response scores (Y; 0 = zero movement, 1 = mild movement, 2 = moderate movement, 3 = severe movement) to sham castration or castration at different stages of the procedure.** Stage: 1 = excision of scrotum; 2 = extrusion of right testis and spermatic cord; 3 = severing of right spermatic cord; 4 = extrusion of left testis and spermatic cord; and 5 = severing of left spermatic cord. ^a,b,c^ Stages with different superscripts differ significantly at *p* ≤ 0.05. A significant effect was found (*p* < 0.001).

There was no significant effect of body weight (*F* = 0.14; d.f. = 30, 485; *p* = 0.99). There was a significant effect of treatment (*p* < 0.001), with SHAM calves having significantly lower scores than all other calves and LIG calves having significantly lower scores than VAPO calves. Probabilities of calves having the lowest score (zero) for treatments SHAM, CAST, LIG and VAPO were 0.87, 0.20, 0.34 and 0.12, respectively (Figure 1). SHAM calves were 9.1 (95% CI: 3.3–25.1), 5.7 (95% CI: 2.1–15.1) and 17.3 (95% CI: 6–50) times more likely to have lower scores than CAST, LIG and VAPO calves, respectively. LIG calves were 3.1 (95% CI: 1.3–7.3) times more likely to have lower scores than VAPO calves. There was a significant effect of stage of procedure (*p* < 0.001), with calves having significantly greater scores in response to stage 2 than to all other stages and calves having significantly greater scores in response to stage 4 than to stage 5. Probabilities of calves having the greatest score (three) in response to stages 1, 2, 3, 4 and 5 were 0.05, 0.22, 0.05, 0.09 and 0.04, respectively (Figure 2). Calves were 5.0 (95% CI: 2–12.4), 4.3 (95% CI: 1.8–10.4), 2.2 (95% CI: 1–5.3) and 5.7 (95% CI: 2.3–13.6) times more likely to have greater scores in response to stage 2 than to stages 1, 3, 4 and 5, respectively. Calves were 2.5 (95% CI: 1.1–5.9) times more likely to have greater scores in response to stage 4 than to stage 5.

### 3.2. Ocular Temperature

There were no significant effects of treatment or body weight (*p* = 0.739 and *p* = 0.479, respectively). There were significant effects of time-point and ambient temperature (*p* = 0.002 and *p* < 0.001, respectively). Maximum ocular temperature was greater at time-point 3 (38.69 ± 0.09 °C) than at time-points 1 and 2 (38.44 ± 0.09 °C and 38.49 ± 0.09 °C, respectively). A low positive relationship between ambient temperature and maximum ocular temperature was identified (R = 0.30).

## 4. Discussion

This study investigated the use of a topical vapocoolant spray to provide local anaesthesia during surgical castration of calves, with results suggesting it is not an adequate form of pain relief for such a procedure. The results of this study also add to information on the efficacy of lignocaine (20 mg/mL) for surgical castration of calves, demonstrating minimal effects during the procedure following administration by the technique used 5 min before the procedure was performed. This study evaluated pain associated with different stages of the surgical castration operation in calves, with extrusion of the testes and pulling of spermatic cords eliciting the greatest pain response. In this study, conclusions are based on subjective behavioural scores only, as although ocular temperature was also measured, changes to this physiological variable were considered due to factors independent of pain.

Descriptive and numerical behavioural scoring systems have previously been used to assess pain associated with ear tagging and ear notching [15], hot-iron disbudding [22], dehorning [23] and castration [17,18,19,20,21] in cattle. In this study, behavioural scoring showed that SHAM calves reacted least to treatment, with a strong probability (0.87) of displaying no response (score zero). This differs from all castrated calves, with probabilities of displaying no response between 0.12 and 0.34 (Figure 1). This was an expected finding which indicates that increasing behavioural scores correspond with increasing degrees of distress and pain. There was no difference between behavioural scores of CAST calves and either LIG or VAPO calves. However, there was a difference between those of LIG and VAPO calves, with LIG calves 3.1 times more likely to have lower scores than VAPO calves. It is possible that lignocaine may have induced a minimal degree of local anaesthesia and vapocoolant spray may have heightened the behavioural response to castration. A previous study investigating the efficacy of a vapocoolant spray for the relief of distress caused by pediatric immunisation found similar results. Children that received vapocoolant demonstrated stronger distress related behaviours towards immunisation compared to children that did not receive vapocoolant [16]. An explanation for findings such as these is that vapocoolant spray may have caused an irritating effect that offset any benefit of pain relief. Another explanation is that immediate prior application of vapocoolant spray may have drawn the attention of subjects to the procedure, heightening the distress response [16]. In addition, it is possible that application of vapocoolant spray to visceral tissue, as in the current study, may have a different sensory effect compared to application of vapocoolant spray to cutaneous tissue, as in previous studies [9,10,11,12,13,14,15,16].

Efficacy of lignocaine (20 mg/mL) for post-operative pain relief following castration of cattle has been investigated in numerous studies with various findings [17,24,25,26,27,28,29,30,31,32]. However, there are fewer studies that have assessed the efficacy of lignocaine (20 mg/mL) specifically for intra-operative pain relief during castration of cattle [17,28,30,32,33,34,35] (Table 1). Of these studies, pain during castration has been assessed using electroencephalographic responses [33], ocular temperature responses [34], cardiovascular responses [33,34], testicle retraction responses [35] and behavioural responses [17,28,30,32] to the procedure. One of these studies showed no effect of caudal lignocaine epidural anaesthesia with epinephrine on measured responses to castration [30]. In all other studies, lignocaine ameliorated responses to castration [17,28,32,33,34,35]. There are multiple methodological differences between the current study and these previous studies, including breed and age of cattle, castration method, timing and technique of lignocaine administration, volume of lignocaine administered and pain assessment outcomes (Table 1). This may explain the contrasting conclusions of these previous studies [17,28,32,33,34,35] and the current study regarding the efficacy of lignocaine for castration of cattle. As in some of these previous studies [17,33,35], in the current study, lignocaine was administered 5 min prior to castration. This is a more realistic representation of its application during routine husbandry procedures performed on cattle in a commercial setting where additional time associated with analgesic administration is impractical [2]. It is possible that the timing and technique of lignocaine administration, including the volume administered, or a combination of these factors resulted in a minimal effect on the behavioural response of calves during surgical castration. It is possible that administration of lignocaine at a higher dose rate could ensure a faster onset of action [7]. This is worthy of investigation in future studies. However, the greater risk of toxicity and nerve damage with increased dose rates of lignocaine is a potential limitation [7].

Vapocoolant spray was investigated in the current study, as it offers a practical method of potentially providing local anaesthesia prior to painful procedures. Anaesthetic efficacy has been achieved using vapocoolant sprays prior to cosmetic botulinum injections [13], intradermal anaesthetic injection [10], venipuncture [11] and vaccination in humans, arthrocentesis in horses [9] and ear tagging and notching in calves [15]. In calves, a vapocoolant spray cooled ear tissue to <10 °C (temperature threshold required for anaesthesia) for 16 s when applied for 3 s and resulted in lower behavioural pain responses to ear tagging and ear notching [15]. In the current study, the same vapocoolant spray was also applied for 3 s to the scrotum and spermatic cords. Application of the spray did not lower the behavioural responses of calves to surgical castration. Similarly, other studies have found no effect of vapocoolant sprays on pain response to intravenous cannulation [36] and skin tests [37] in humans and jugular catheterisation in horses [9]. The effect of cooling can differ in relation to the type, length and depth of nerve fibres and the degree of tissue vascularisation [15]. The variation between studies could be attributed to the type of vapocoolant, duration of spray time and the type and location of tissue injury involved with each procedure. It is recognised that products with short application durations (≤3 s) are most practical for use during routine husbandry procedures performed in a commercial farm setting. For this reason and on the basis of the results from the previous study on ear tagging and ear notching [15], a 3-second spray was investigated in the current study. Even if a longer spray would have induced anaesthesia, the pain associated with the pulling stages of castration would still not be alleviated, as this involves sensory responses along the length of the spermatic cords, into the inguinal canal and potentially visceral pain centres during externalisation of the tissues [38]. As these stages of the procedure were identified as the most painful in the current trial, vapocoolant sprays appear ineffective for use during surgical castration of calves.

This study presents on behavioural responses, as a measure of pain, associated with the different stages of castration in beef calves. Procedural sources of pain during surgical castration have been identified in piglets through analysis of vocal responses [38] where results are similar to those of the current study (Figure 2). In piglets, initial restraint, washing of the ano-genital area, incision of the scrotum, and pulling/incision of the spermatic cords were all compared, with pulling/incision of the spermatic cords evoking the greatest vocalisation responses, as measured in the rate of high frequency calls [38]. Scrotal excision and pulling/severing of the spermatic cords affect different tissues, cutaneous and visceral, respectively [31]. Typically, visceral tissues are less sensitive to pain than non-visceral tissues [39]. Visceral pain is usually dull, diffuse and poorly localised compared to sharp, well localised somatic pain [40]. However, the testes are among the few viscera producing sharp, localised pain due to well innervated tissue [38] and the presence of true nociceptors [39]. Visceral pain can result from non-damaging stimuli such as distension or traction [38] and is often associated with exaggerated autonomic reflexes [41]. Pulling of the spermatic cords likely results in sensation along the length of the spermatic cords and into the inguinal canal and beyond, likely resulting in greater pain than the rapid excision and severing stages of the castration procedure [38]. This may explain why the probability of calves displaying the most severe response (score three) was greatest for stage 2 (extrusion of the first spermatic cord) (0.22) and followed by stage 4 (extrusion of the second spermatic cord (0.09), in the current study. There appeared to be an effect of the order of stages, with calves being 2.2 times more likely to have greater behavioural scores to extrusion of the first spermatic cord (stage 2) than to extrusion of the second spermatic cord (stage 4). Similarly, the last stage of the procedure resulted in the least severe behavioural response overall, with a low probability (0.04) of calves having a score three (Figure 2). Behavioural responses of lame sows to thermal or pressure algometry of the rear legs have been shown to differ according to the leg (right or left) that was first tested. The right leg, tested first, was shown to tolerate less mechanical pressure or thermal stimulation than the left leg. This difference in nociceptive threshold was probably due to the sows being startled by the first manipulation [42]. This could be the case in the current study, with the pain associated with pulling the first spermatic cord likely startling calves and therefore resulting in a greater behavioural response.

Measurement of ocular temperature has been used to evaluate stress and pain associated with disbudding [43,44,45] and castration [34,46] of calves. A decrease in ocular temperature following stress or pain is possibly due to vasoconstriction of capillary vessels caused by activation of the sympathetic nervous system. A subsequent increase in ocular temperature could be the result of increased dominance of the parasympathetic nervous system which results in vasodilation of blood vessels [47]. In this study, the increase in ocular temperature following castration or sham castration could not be attributed to the experience of pain, as there was no significant difference between SHAM and CAST calves. These results contrast from those of some previous studies where differences in ocular temperature have been detected between control calves and calves undergoing disbudding [36,37] or castration [34]. However, as in the current study, other previous research has found no difference in ocular temperature between control calves and calves undergoing disbudding [45] or castration [46]. Where no difference has been found between control and castrated calves, the methodology involved capturing a series of photographs before, during and after treatment [46], similar to the current study. Whereas a difference has been detected when continuous recordings of ocular temperature were collected every 20 s for 10 min prior to treatment and 20 min post treatment [34]. Another difference in methodology refers to the exact location of where maximum temperature was detected, which was either the whole eye [46] or the medial posterior palpebral border of the lower eyelid [34]. Albeit a weak correlation, the current study found a positive relationship between ambient temperature and maximum ocular temperature, despite calibration of the infrared camera for atmospheric conditions. The effect of ambient temperature on ocular temperature has not been examined in previous studies [34,46] and should be considered in future research. Habituation of calves to handling facilities and restraint was not conducted in the current study and it appears that stress, rather than pain, had the dominant effect on ocular temperature, which has also been previously suggested [45] and should be considered for future studies.

## 5. Conclusions

The results of the current study showed that vapocoolant spray applied for 3 s to the scrotum and each spermatic cord during surgical castration of beef calves did not reduce behavioural responses of the animals to the procedure. Lignocaine (20 mg/mL), administered as 3 mL injected into each side of the scrotum and a further 3 mL into each spermatic cord 5 min prior to castration, also did not significantly reduce behavioural responses of animals during the procedure. Therefore, this study could not demonstrate that adequate intra-operative anaesthesia for surgical castration of beef calves was provided by either of the interventions tested, as assessed by scoring the behavioural response of the animals on a numerical rating scale. Therefore, the interventions evaluated in this study are not recommended for inclusion in a practical analgesic regime for addressing peri-operative pain of castration in calves. Future research should consider analgesic interventions that address the pain associated with pulling of the spermatic cords, as this stage of the surgical castration procedure appears to be the primary source of intra-operative distress in beef calves.

## Figures and Tables

**Table 1:** Methodology in previous studies investigating the efficacy of lignocaine HCl (20 mg/mL) for pain relief during castration of cattle.

Study	Breed and Age of Calves	Timing and Technique of Lignocaine Administration	Castration Method	Outcomes Used for Assessment of Intra-operative Pain
Melendez et al. 2018 (28)	Angus crossbreed; 7–8 months	5 mL with epinephrine into each spermatic cord and 20 mL subcutaneously around the neck of the scrotum 30 min prior to castration	Surgical with crushing of spermatic cords using an emasculator	Behavioural responses (visual analog scale, distance of head movement, frequency of leg movements, acceleration force and head gate force)
Neves et al. 2017 (35)	Mixed breed Nellore; 28–40 months	3 mL subcutaneously at each site of scrotal incision and 4 mL into each spermatic cord 5 min prior to castration	Surgical	Testicle retraction responses (scored as positive or negative)
Lehmann et al. 2017 (33)	*Bos indicus*; 6–8 months	Approximately 6 mL into each testicle and 2 mL subcutaneously into scrotal skin 5 min prior to castration	Surgical	Electroencephalographic and cardiovascular responses
Stewart et al. 2010 (34)	Friesian; 4 months	5 mL into each testicle through the distal pole and into the distal end of the scrotum followed by a subcutaneous infiltration (7 mL) into the neck of the scrotum 10 min prior to castration	Surgical	Ocular temperature, cardiovascular and catecholamine responses
Currah et al. 2009 (30)	Angus crossbreed; 3 months	0.06 mg/kg BW with epinephrine injected into the sacrococcygeal epidural space 39 to 88 min prior to castration	Surgical	Behavioural responses (number of vocalisations and exertion force against the restraint)
Boesch et al. 2008 (32)	Holstein Friesian, Brown Swiss, Holstein Friesian beef crossbred, Brown Swiss beef crossbreed; 2–7 days	2 mL proximally into each spermatic cord and 1.5 mL each at the cranial and caudal aspects subcutaneously on both sides of the scrotal neck 20 min prior to castration	Burdizzo	Behavioural response (frequency of struggling events)
Thuer et al. 2007 (17)	Simmental, Simmental cross Red Holstein; 21–28 days	10 mL into both spermatic cords and subcutaneously around the scrotal neck 5 min prior to castration	Burdizzo or rubber ring	Behavioural response (scored on a numerical rating scale of 0–3)
